# Prolonged Effects on Frontline Caregivers: Occupational Stress and Mental Well-Being in Transformed Healthcare Environments Post-COVID-19

**DOI:** 10.3390/ijerph23020271

**Published:** 2026-02-22

**Authors:** Rauer Ferreira Franco, Jefferson Martinelli, Amanda Oliva Spaziani, Luis Carlos Spaziani, João Daniel de Souza Menezes, Matheus Querino da Silva, Emerson Roberto dos Santos, Rita de Cássia Helú Mendonça Ribeiro, Josimerci Ittavo Lamana Faria, Janaína Aparecida de Sales Floriano, Fernando Nestor Facio Júnior, Nádia Antônia Aparecida Poletti, Flávia Cristina Custódio, Clarissa Albuquerque Vaz Nunes, Franciane Michele da Silva, Maysa Alahmar Bianchin, Luís Cesar Fava Spessoto, Ana Paula Bernardes da Rosa, Maria Helena Pinto, Cíntia Canato Martins, Marli de Carvalho Jerico, Fabiana de Souza Orlandi, Lais Fernanda de Amorin, Paula Buck de Oliveira Ruiz, Fabricio Sidnei da Silva, Luan Souza do Nascimento, Catia Canova Fraccari, Gerardo Maria de Araújo Filho, Marcia Regina Furlani, Stela Regina Pedroso Vilela Torres de Carvalho, Ana Maria Rita Pedroso Vilela Torres de Carvalho Engel, Thiago Sivieri, Bruna Santos de Oliveira Martins, Daniela Gonçalves Faustino, Maicon José de Jesus Vijarva, Júlio César André

**Affiliations:** 1 Center for Studies and Development of Health Education—CEDES, São José do Rio Preto Medical School—FAMERP, São José do Rio Preto 15090-000, Brazil; rauerf@hotmail.com (R.F.F.); emerson.santos@edu.famerp.br (E.R.d.S.); josimerci.faria@edu.famerp.br (J.I.L.F.); janainaflorianoadv@gmail.com (J.A.d.S.F.); fernando.nestor@famerp.br (F.N.F.J.); fccustodio@funecsantafe.edu.br (F.C.C.); clarissaavaz@gmail.com (C.A.V.N.); franciane.ata@gmail.com (F.M.d.S.); maysa@famerp.br (M.A.B.); cintiacanato1@gmail.com (C.C.M.); marli@famerp.br (M.d.C.J.); catiacanovaf@gmail.com (C.C.F.); marciarelani@hotmail.com (M.R.F.); stela.carvalho@edu.famerp.br (S.R.P.V.T.d.C.); ana.engel@edu.famerp.br (A.M.R.P.V.T.d.C.E.); sivierithiago@gmail.com (T.S.); bsb.oliveira@gmail.com (B.S.d.O.M.); danielagf17@gmail.com (D.G.F.); maiconvijarva@yahoo.com.br (M.J.d.J.V.); julio.andre@edu.famerp.br (J.C.A.); 2 Brazil University, Fernandópolis, São Paulo 15613-899, Brazil; spazianimedicina@gmail.com (A.O.S.); prof.fabricio@ymail.com (F.S.d.S.); nascimentoluansouza@gmail.com (L.S.d.N.); 3 Federal University of the State of Rio de Janeiro, Rio de Janeiro 21941-617, Brazil; jeffmartinelli@hotmail.com; 4 Fernandópolis Educational Foundation, Fernandópolis, São Paulo 15608-380, Brazil; luiscspaziani@gmail.com; 5 São José do Rio Preto Medical School—FAMERP, São José do Rio Preto 15090-000, Brazil; matheus.querino@edu.famerp.br (M.Q.d.S.); lcspessoto@gmail.com (L.C.F.S.); mariahelena@famerp.br (M.H.P.); filho.gerardo@gmail.com (G.M.d.A.F.); 6 General Nursing Department, São José do Rio Preto Medical School—FAMERP, São José do Rio Preto 15090-000, Brazil; ritadecassia@famerp.br (R.d.C.H.M.R.); nadia@famerp.br (N.A.A.P.); 7 UNORTE—University Centre of Northern São Paulo, São José do Rio Preto 15020-040, Brazil; paulabernardes@unorte.edu.br; 8 Federal University of São Carlos, São Carlos 13565-905, Brazil; forlandi@ufscar.br; 9 Faculty Educational Group—UNITERP, São José do Rio Preto 15091-260, Brazil; lais.fernanda@uniterp.com.br (L.F.d.A.); paula.buck@uniterp.com.br (P.B.d.O.R.)

**Keywords:** COVID-19, occupational stress, mental health, quality of life, emergency nursing

## Abstract

**Highlights:**

**Public health relevance—How does this work relate to a public health issue?**

- This study investigates the mental health and quality of life among nursing technicians working in Brazilian Unified Health System (SUS) emergency units, a critical workforce for public health, particularly in the post-COVID-19 pandemic context.
- The research demonstrates that the inherently stressful work environment and the psychosocial sequelae of the pandemic contribute to pervasive anxiety and compromise the quality of life and social relationships of these professionals, thereby directly impacting their capacity to deliver high-quality healthcare to the population.

**Public health significance—Why is this work of significance to public health?**

- The study reveals that the intrinsic and demanding nature of emergency work, exacerbated by the prolonged effects of the pandemic, represents the primary stressor for nursing technicians, superseding sociodemographic and professional factors such as salary or workload. This indicates a systemic public health issue requiring environment-centric approaches.
- The identified widespread anxiety, characterized as a common and persistent experience among these professionals, suggests a “chronic allostatic load” or a “Long COVID-19” effect on the mental health of frontline workers. This condition can precipitate compassion fatigue and care errors, ultimately jeopardizing patient safety and the effectiveness of SUS emergency services.

**Public health implications—What are the key implications or messages for practitioners, policy makers and/or researchers in public health?**

- For policymakers and healthcare administrators, support interventions must extend beyond traditional solutions (e.g., salary adjustments or workload modifications), emphasizing the adaptation of work environments to the “new realities” of the post-pandemic era, fostering communication, and combating isolation.
- It is imperative to develop comprehensive strategies that enhance the overall quality of life and coping mechanisms of nursing technicians through robust organizational and individual support. This approach aims to mitigate amplified psychological impacts and ensure the sustained quality and safety of patient care in the post-pandemic landscape.

**Abstract:**

Objectives: This study evaluated Quality of Life (QoL) and mental health among nursing technicians in Brazilian Unified Health System (SUS) emergency units, specifically exploring occupational safety and well-being in the post-COVID-19 era. Design: A quantitative, descriptive, exploratory, cross-sectional design was employed. Methods: Data from 146 nursing professionals in Brazilian SUS emergency units were collected remotely during the post-acute pandemic phase (July–Nov 2024). QoL (WHOQOL-BREF) and mental health (HADS) were assessed, followed by descriptive and correlational statistics. Results: The predominantly female, experienced sample showed heterogeneous general QoL but pervasive anxiety, reflecting a sustained psychological burden. Sociodemographic/professional factors had a negligible impact; the emergency environment’s overwhelming influence, intensified by post-pandemic challenges, was key. Psychological distress was strongly negatively correlated with overall QoL and depression in the social domain. Conclusions: The intrinsic nature of emergency work, amplified by persistent psychosocial effects of the global health crisis, drives anxiety and impairs QoL/social relationships. Interventions strengthening QoL, enhancing coping, and adapting work environments to new realities (e.g., loneliness, prolonged mental health impacts) are vital for professional well-being and patient care in this post-pandemic era.

## 1. Introduction

Quality of Life (QoL) is a multidimensional construct encompassing physical, psychological, social, and environmental aspects of human experience, transcending the mere absence of disease [1]. The World Health Organization (WHO) defines it as “an individual’s perception of their position in life in the context of the culture and value systems in which they live and in relation to their goals, expectations, standards and concerns” [2]. In the field of nursing, QoL acquires specific characteristics influenced by work environment conditions, occupational tensions, and the challenge of balancing personal and professional spheres [3]. In Brazil, the nursing professional category represents the largest contingent of professionals in the Unified Health System (SUS), being fundamental for the implementation of care actions [4]. However, the current scenario, marked by population aging and increasing demand for health services, has led to work overload and burnout [5]. In emergency and urgent care services, the performance of these professionals is characterized by high operational tension, unpredictable demands, and exposure to traumatizing events [3,4,5,6,7,8,9,10].

The mental health of nursing professionals directly influences the quality of care provided [10,11]. Professional exhaustion compromises the capacity for effective communication and empathy, potentially leading to errors and a perception of more impersonal care [12,13]. The recent global health crisis, triggered by the COVID-19 pandemic, acted as a catalyst and amplifier of these pre-existing tensions, casting a prolonged shadow over the occupational safety and mental health of healthcare workers worldwide. Although the acute phase of the pandemic may have subsided, its profound and lasting impact, often termed “Long COVID-19” in reference to persistent health consequences, continues to affect these professionals. This impact extends beyond physical symptoms, encompassing a persistent psychological burden, manifested by a significant increase in stress, elevated anxiety, feelings of loneliness and isolation, and a critical need for enhanced coping strategies and self-care. The pandemic fundamentally reconfigured work environments, from the acceleration of teleworking and online work implementation to alterations in communication and socialization opportunities, vital resources for mental health. Understanding these “new changes” and the extent to which work environments will never return to their pre-COVID-19 states is of paramount importance. The investigation of QoL among nursing technicians in the SUS is, therefore, a strategic necessity, as satisfied and healthy professionals offer high-quality care [9,10,11,12,13,14,15,16].

Work conditions in emergency settings significantly impact the physical dimension, with a prevalence of musculoskeletal disorders and sleep pattern alterations [6,7], and the emotional dimension, with significant symptoms of anxiety and depression correlated with occupational stress [8,9]. Interventions aimed at promoting resilience and self-care among nurses result in improved quality of reception and humanization of care [14,15].

Despite the growing scientific production, the literature still presents significant gaps. Many studies have a cross-sectional design, limiting the understanding of causal relationships and the temporal dynamics between organizational factors and health outcomes [17,18]. There is also a scarcity of studies employing mixed methods and a geographical concentration in large centers, which obscures the reality of more precarious contexts. Filling these gaps is imperative, especially in the face of the impacts of the COVID-19 pandemic on professionals’ mental health [19] and the progressive global deficit of nursing professionals [20], which makes the investigation of factors promoting professional retention and satisfaction urgent. Promoting the quality of life of these professionals is not just an occupational health issue but a strategic element for qualifying and humanizing care in critical services [21].

**Figure 1:**
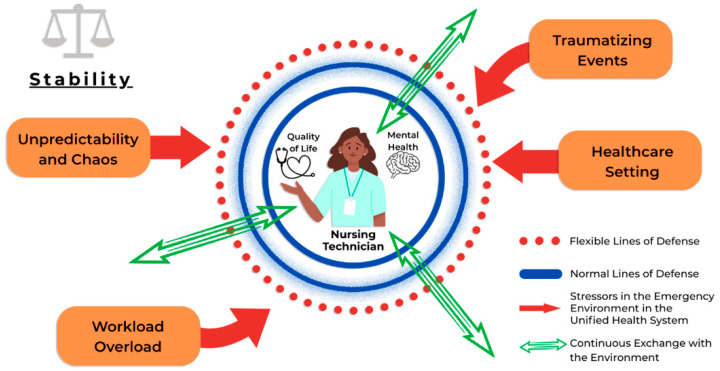
Conceptual representation of Betty Neuman’s Systems Model applied to nursing technicians in emergency healthcare settings within the Unified Health System (SUS). Source: This figure is original to the authors.

In this context, Betty Neuman’s Systems Model offers an adequate theoretical lens for understanding the experience of nursing professionals. This theory conceives the human being as an integral system in continuous interchange with the environment, seeking to maintain its stability. The intense demands of emergency work in the SUS can be understood as ‘stressors’ that threaten the ‘lines of defense’ of nursing technicians, directly impacting their quality of life and mental health (Figure 1) [22,23].

The diagram illustrates the nursing technician as a central client system encompassing quality of life and mental health dimensions, surrounded by protective lines of defense (normal and flexible). Environmental stressors characteristic of emergency healthcare contexts, including workload intensity, unpredictability, traumatic exposures, and systemic healthcare pressures, are depicted as external forces that penetrate these defensive barriers. The bidirectional arrows represent the continuous dynamic exchange between the individual system and the healthcare environment, demonstrating how occupational stressors can compromise system stability and ultimately affect the professional’s well-being and mental health outcomes.

Therefore, this study aimed to evaluate the quality of life and mental health of nursing technicians working in emergency settings within the Unified Health System (SUS).

This study aimed to evaluate the quality of life and mental health of nursing technicians working in emergency settings within the Unified Health System (SUS), with a particular lens on how these factors are modulated by the prolonged challenges of occupational safety and mental health, precipitated or intensified by the COVID-19 pandemic, thereby contributing to an in-depth understanding of the persistent post-pandemic impacts on this essential workforce.

## 2. Materials and Methods

### 2.1. Design

The present study adopted a quantitative approach, with a descriptive, exploratory, and cross-sectional design.

### 2.2. Study Setting and Sampling

Data collection was conducted remotely through an electronic form created on the Google Forms platform and digitally distributed via email and social media (WhatsApp^®^, Facebook^®^, and Instagram^®^). The study participants were nursing technicians actively working in emergency and urgent care units linked to the Unified Health System (SUS) in the municipalities of Fernandópolis or Votuporanga, São Paulo state, and having a minimum professional experience of six months in the aforementioned role. These municipalities, located in the northwest of São Paulo, have comparable geographical and demographic characteristics, presenting similar Human Development Indexes (HDI) [24]. The target population consisted of nursing technicians working in these cities [25]. Of the 150 professionals invited to participate, 146 adhered to the study, while 4 did not respond to the requests. This represents a high response rate of 97.3%. Although an explicit statistical power calculation for sample size justification was not detailed, the participation of 146 professionals represented a high response rate from the invited population. This study is exploratory in nature, and thus, a formal a priori power calculation for sample size was not performed; however, the high response rate from the target population strengthens the representativeness of the assessed group within these specific municipalities.

### 2.3. Instruments

For the assessment of quality of life, the WHOQOL-BREF 2 was chosen. This is a concise instrument developed by the WHOQOL Group of the World Health Organization (WHO). The WHOQOL-BREF consists of 26 questions, with two general questions about quality of life and satisfaction with health, and the others distributed across four broad domains: Physical, Psychological, Social Relationships, and Environment. The Brazilian version of the WHOQOL-BREF was translated, adapted, and validated in the country by Fleck et al. (1999) [26], demonstrating satisfactory psychometric qualities for use in research with the Brazilian population.

Additionally, to assess anxiety and depression levels, the Hospital Anxiety and Depression Scale (HADS) was used [27]. The HADS consists of 14 items, equally distributed into two seven-item subscales: Anxiety Subscale (HADS-A) and Depression Subscale (HADS-D). The suitability of the HADS for the Brazilian context is confirmed by its translation and validation. Initial studies such as [28], followed by the work considered pioneering by Botega et al. (1995) [29], which demonstrated the internal consistency and construct validity of the scale in a sample of hospitalized patients, reinforced its psychometric robustness for the Brazilian population. HADS is widely recognized and used beyond the hospital environment, being successfully applied in various other contexts, including primary care, occupational health, epidemiological, and general population studies [30,31].

### 2.4. Data Collection

Data collection was conducted remotely from July to November 2024. Participants accessed the questionnaire through an electronic form (Google Forms), distributed via email and social media (WhatsApp^®^, Facebook^®^, and Instagram^®^). Prior to participation, all individuals were presented with a Free and Informed Consent Form (FICF). They were required to read and explicitly accept its terms digitally before gaining access to the questionnaire, ensuring informed consent was obtained. Participants then completed the questionnaire autonomously and remotely. Upon completion, a confirmation of participation, including a copy of the FICF and their responses, was automatically sent to their email [32]. This procedure ensured transparency and documented consent for all participants.

### 2.5. Data Analysis

Data analysis was conducted in four main stages: calculation of factorial scores, exploratory analysis, group comparison, and correlation analysis. Initially, factorial scores were estimated from models with latent variables, considering that ordinal responses to the instruments reflect underlying continuous psychological attributes. The Empirical Bayes Modal method with whitening and coloring transformations was used to preserve correlations between factors, as recommended by Kessy (2018) [33].

Exploratory analysis involved descriptive statistics (mean, standard deviation, percentiles, minimum and maximum values for numerical variables, and frequencies for categorical variables), in addition to normality tests (Shapiro–Wilk) and assessment of skewness and kurtosis. For comparison between groups in continuous variables, the Mann–Whitney U test was applied, with effect size calculated using R-biserial correlation. Correlations between continuous variables were examined using Pearson’s correlation coefficient, with attenuation corrected for measurement error, as guided by Metsämuuronen (2022) [34].

The *p*-value, 95% confidence intervals, and the squared coefficient as an effect size measure were reported. Analyses were performed using R software (v. 4.4.1) with the Hmisc, psych, and lavaan packages. *p*-values less than 0.05 were considered statistically significant [35]. All data supporting the findings of this study have been made publicly available through the Open Science Framework (OSF) repository and can be accessed at: https://osf.io/7q8zw/.

### 2.6. Ethical Considerations

All ethical precepts established in Resolution No. 466/2012 of the National Health Council 32, which provides guidelines and regulatory norms for research involving human beings, were fully respected. The project was submitted for approval to the Research Ethics Committee (CEP) of the Faculty of Medicine of São José do Rio Preto (FAMERP), having obtained a favorable opinion under protocol number 6.918.669. Participants were duly informed about the objectives, procedures, risks, and benefits of the research, and only after reading the full FICF could they access and answer the instruments. The term emphasized freedom of participation and the right to withdraw at any stage of the study, without any prejudice. Cybersecurity measures, such as data encryption and anonymization, were implemented to protect the privacy and integrity of the information, and the data will be destroyed after the study’s conclusion.

## 3. Results

### 3.1. Sociodemographic and Professional Variables

The study sample consisted of 146 nursing technicians. The target population comprised 150 nursing technicians, of whom 146 adhered to the study, and 4 did not respond to requests. The predominant profile was female, representing 86% of the sample, with a mean age of 36.8 years, reflecting a professional group in the active phase of their career. The geographical distribution revealed that most participants (84%) resided and worked in the same municipality. Regarding employment ties, the vast majority (83%) had only one professional tie. The most common work regimen was 12 h of work followed by 36 h of rest (89% of participants), a characteristic pattern of emergency and urgent care units. Professional experience demonstrated significant time in the field, with 36% of the sample having between 10 and 20 years of experience. Regarding salary range, most professionals (67%) earned between R$ 3000.00 and R$ 4000.00.

The complete descriptive data of the sample are presented in Table 1.

### 3.2. Quality of Life Analysis

Quality of life analysis was performed using standardized factorial scores from the WHOQOL-BREF. It is important to note that for these scores, positive values indicate levels above the sample mean in the evaluated construct, while negative values indicate levels below the mean.

The descriptive analysis of scores revealed distinct patterns in the perception of quality of life among participants. The “General Quality of Life” domain demonstrated the greatest heterogeneity in responses, suggesting a diversity of individual perceptions regarding global well-being. In contrast, the “Environment” domain showed the most homogeneous distribution of scores, indicating a consensus among professionals regarding the environmental aspects influencing their quality of life. The “Social Relationships” and “Physical” domains exhibited intermediate variability in their distributions.

Despite differences in dispersion, the medians of all domains remained close to zero, pointing to a balanced central tendency in the perception of the general quality of life of the sample. Regarding data normality, only the “Environment” domain showed a distribution compatible with the Gaussian pattern. The other domains (General Quality of Life, Social Relationships, and Physical) demonstrated non-parametric distributions, relevant information that guided the choice of subsequent statistical tests.

The complete statistical details of the WHOQOL-BREF factorial scores are presented in Table 2.

**Figure 2:**
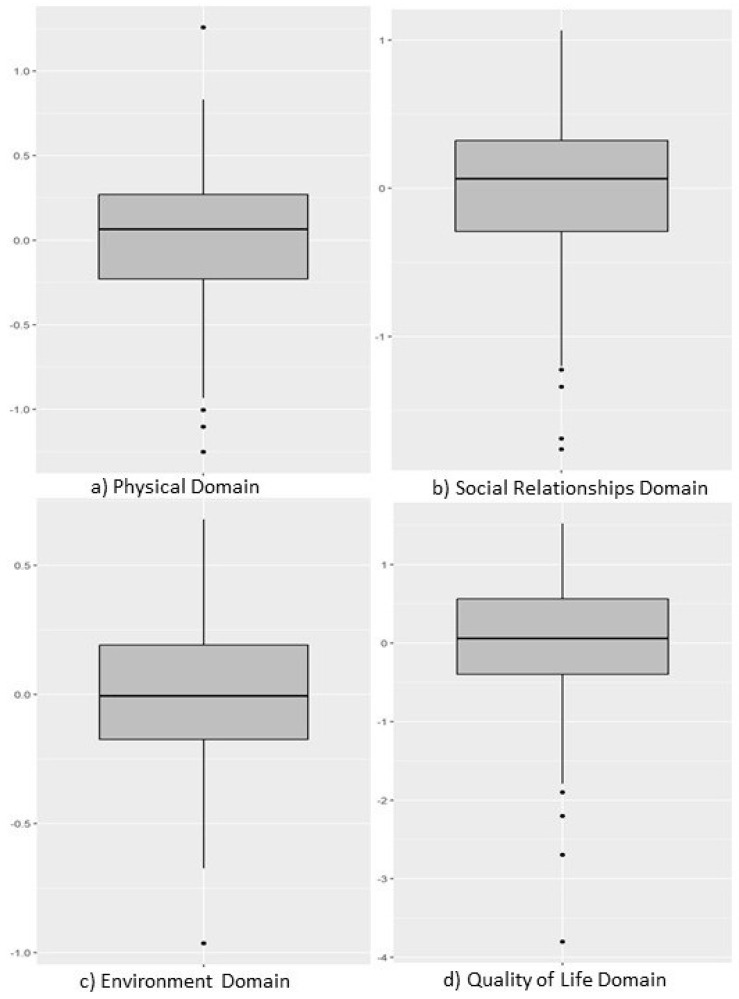
Histogram distribution of WHOQOL-BREF factorial scores for the: (**a**) physical domain variable; (**b**) social relationships domain; (**c**) environment domain; (**d**) quality of life domain. (n = 146; São Paulo—Brazil; 2025). Source: Author.

**Figure 3:**
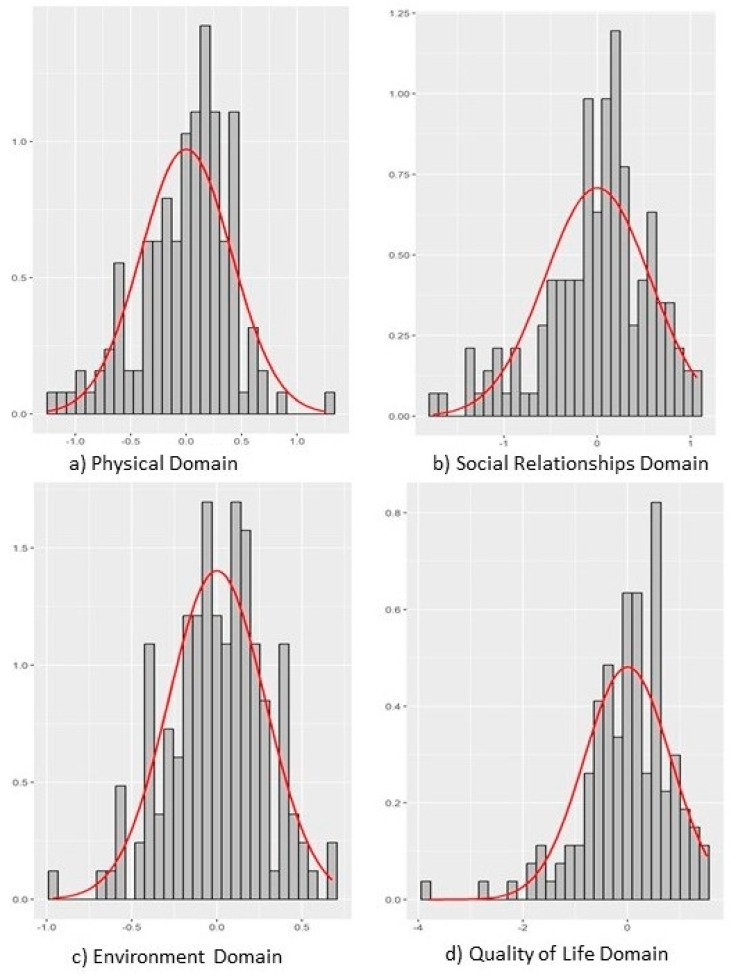
Boxplot distribution of WHOQOL-BREF factorial scores for the: (**a**) physical domain variable; (**b**) social relationships domain; (**c**) environment domain; (**d**) quality of life domain. (n = 146; São Paulo—Brazil; 2025). Source: Author.

Figure 2 and Figure 3 present the distribution of scores for each of the latent variables related to quality of life modeled in the sample.

Note for Figure 2 (Boxplot): To enhance clarity, the box plot in Figure 2 can be modified to display actual values within the boxes, such as the median and interquartile range (IQR), if not already present. The figure legend should be updated accordingly to reflect these numerical representations.

### 3.3. Mental Health Analysis

The mental health of professionals was assessed using the Hospital Anxiety and Depression Scale (HADS). Standardized factorial scores revealed that psychological distress stood out as the domain with the greatest heterogeneity in the sample, indicating substantial variations in the subjective experiences of psychological suffering among participants, possibly reflecting different levels of coping with work demands.

The domain of depressive symptoms showed intermediate dispersion. In contrast, the “Anxiety” domain demonstrated the lowest variability, suggesting that anxiety may be a widely shared experience among nursing technicians working in high-complexity contexts, given the intrinsically stressful nature of these environments.

Medians close to zero across all domains indicated a balanced central tendency in the overall distribution of scores. Normality analysis showed that the anxiety and depressive symptoms domains did not follow a normal distribution, while psychological distress exhibited behavior closer to normality. These findings were crucial for grounding the choice of non-parametric statistical tests in subsequent inferential analyses.

Detailed statistical data for the HADS factorial scores are presented in Table S1 of the Supplementary File.

Figure S1 in the Supplementary File presents the distribution of scores for each of the latent variables related to the HADS in the sample.

### 3.4. Comparative Analysis of the WHOQOL-BREF Quality of Life Instrument

#### 3.4.1. Comparison of WHOQOL by Place of Residence

The comparative analysis of WHOQOL-BREF scores between nursing technicians residing in Fernandópolis and Votuporanga revealed specific distinctions in the perception of quality of life. Professionals from Votuporanga showed slightly higher scores in the “Physical” domain, with a tendency towards statistical significance (*p* = 0.055, which is considered a marginal *p*-value), indicating a modest advantage in the perception of physical well-being in this group.

In the “Social Relationships” and “Environment” domains, no statistically significant differences were observed between the groups, suggesting similar perceptions regarding social support, quality of interpersonal relationships, and environmental conditions in both municipalities.

However, a statistically significant difference was identified in the “General Quality of Life” domain (*p* = 0.045), where residents of Votuporanga reported a slightly more positive global perception. It is important to note that these subgroup comparisons, particularly in the “Physical” domain (*p* = 0.055), are exploratory and performed with limited subgroup sizes, which should be considered when interpreting these findings. These findings provide insights into the contextual variations in perceived quality of life by professionals, as detailed in Table S2 of the Supplementary File.

#### 3.4.2. Comparison of WHOQOL by Salary

The comparative analysis of WHOQOL-BREF scores across different salary groups revealed that monthly remuneration was not a determining factor in the perception of quality of life among nursing technicians in this sample.

In general, no statistically significant differences were observed in any of the evaluated domains (Physical, Social Relationships, Environment, and General Quality of Life) based on salary range. Although some medians might exhibit small variations between income groups (e.g., a slight tendency for higher scores in the physical domain for higher salaries, and vice versa for social relationships), these differences did not reach statistical significance, and effect sizes were classified as very small.

These findings suggest that, in the context of this study, salary level did not substantially influence participants’ perception of quality of life. Complete details on these comparisons are systematized in Table S3 of the Supplementary File.

#### 3.4.3. Comparison of WHOQOL by Workload

The comparative analysis of WHOQOL-BREF scores between different working hour regimens also revealed no statistically significant differences in participants’ perception of quality of life.

Although professionals with lower workloads (grouping 6 h and 8 h daily, N = 16) showed slightly higher medians in domains such as “Physical” and “Social Relationships,” these small variations did not reach statistical significance. Similarly, in the “Environment” domains and the overall evaluation of “Quality of Life,” the differences between groups were considered non-significant. It is important to note that these subgroup comparisons are exploratory and performed with limited subgroup sizes, which should be considered when interpreting these findings.

#### 3.4.4. Comparison of WHOQOL by Professional Tie

The comparative analysis of WHOQOL-BREF scores between professionals with a single professional tie and those with multiple ties revealed, for the most part, an absence of statistically significant differences in the perception of quality of life. This held true for the “Physical,” “Environment,” and “General Quality of Life” domains, where the number of professional ties did not demonstrate a relevant influence.

However, a notable trend was observed in the “Social Relationships” domain: participants with only one professional tie showed higher scores compared to those with two or more ties. Although this did not categorically reach the threshold of statistical significance (*p* = 0.073, a marginal *p*-value), these data suggest a possible association between the number of professional ties and the perceived quality of social relationships.

It is important to note that these subgroup comparisons are exploratory and performed with limited subgroup sizes, which should be considered when interpreting these findings.

#### 3.4.5. Comparison of WHOQOL by Sex

The comparative analysis of WHOQOL-BREF scores between sexes revealed an absence of statistically significant differences in most evaluated domains. The “Physical”, “Environment”, and “General Quality of Life” domains showed similar scores between men and women, indicating that participants’ sex was not a determining factor in the perception of these aspects of quality of life.

Nevertheless, in the “Social Relationships” domain, women demonstrated higher scores than men, showing a tendency towards statistical significance (*p* = 0.069, a marginal *p*-value). Although this difference did not reach categorical significance, the results suggest a possible distinction in the perception of the quality of interpersonal relationships between sexes in this sample. It is important to note that these subgroup comparisons are exploratory and performed with limited subgroup sizes, which should be considered when interpreting these findings.

### 3.5. Comparative Analysis of HADS (Hospital Anxiety and Depression Scale)

#### 3.5.1. Comparison of HADS by Workload

The comparison of Hospital Anxiety and Depression Scale (HADS) scores between different working hour regimens did not reveal statistically significant differences between the groups.

In the “Anxiety” domain, although participants with a workweek of up to eight hours daily (N = 16) showed slightly lower scores, this difference was not statistically relevant. The same pattern of similarity was observed in the “Depression” and “Psychological Distress” domains, where there was no evidence of significant differences related to work regimens.

These findings indicate that workload, as evaluated, did not exert a significant impact on the emotional aspects (anxiety, depression, and psychological distress) of nursing technicians.

#### 3.5.2. Comparison of HADS by Place of Residence

The comparative analysis of Hospital Anxiety and Depression Scale (HADS) scores between nursing technicians residing in Fernandópolis and Votuporanga did not reveal statistically significant differences in the evaluated domains.

In the “Anxiety” domain, scores were similar between groups, indicating that the place of residence did not significantly influence participants’ anxiety levels. Similarly, for the “Depression” domain, although Votuporanga showed slightly lower scores, this difference did not reach statistical significance. The same pattern was observed for overall “Psychological Distress,” where no relevant differences were found.

In summary, these findings suggest that the city of residence (Fernandópolis or Votu-poranga) does not exert a significant impact on the levels of anxiety, depression, or psychological distress of nursing technicians in this sample.

#### 3.5.3. Comparison of HADS by Salary

The comparative analysis of HADS scores among participants in different salary ranges did not reveal statistically significant differences in any of the evaluated domains.

“Anxiety” scores were practically identical between income groups, indicating that remuneration had no relevant impact on anxiety levels. In the “Depression” domain, although there was a slight variation in medians, this difference did not reach statistical significance, reinforcing the absence of a differential effect related to income. Similarly, overall “Psychological Distress” showed very similar scores across different salary strata.

In sum, the results suggest that salary range does not significantly influence the emotional aspects (anxiety, depression, and psychological distress) of nursing technicians as assessed by HADS.

#### 3.5.4. Comparison of HADS by Professional Tie

The comparative analysis of Hospital Anxiety and Depression Scale (HADS) scores between participants with a single professional tie and those with two or more ties did not reveal statistically significant differences in the evaluated domains.

In the “Anxiety” domain, although professionals with multiple ties showed slightly higher scores, this difference did not reach statistical significance. Similarly, “Depression” and “Psychological Distress” scores were comparable between groups, indicating that the number of professional ties did not have a relevant impact on these aspects.

In sum, the results of this sample suggest that the number of professional ties does not significantly affect the levels of anxiety, depression, or psychological distress of nursing technicians.

#### 3.5.5. Comparison of HADS by Sex

The comparison of Hospital Anxiety and Depression Scale (HADS) scores between sexes did not reveal statistically significant differences in the evaluated domains.

In the “Anxiety” domain, men showed slightly higher scores compared to women, but this difference was not statistically significant. Similarly, “Depression” and “Psychological Distress” scores were similar between male and female groups.

In synthesis, the results suggest that participants’ sex did not have a significant impact on the levels of anxiety, depression, or psychological distress in this sample.

### 3.6. Correlation of WHOQOL-BREF with HADS

The Pearson correlation matrix revealed few statistically significant associations among the analyzed variables. As expected from a bifactor model, correlations between physical, social, and environmental domains and the general quality of life were close to zero.

Table S4 in the Supplementary File presents the Pearson correlation matrix.

Analysis of the deattenuated correlation matrix allowed for identifying clearer patterns and more pronounced relationships between variables compared to the original matrix. In this analysis, “Psychological Distress” maintained the strongest negative correlation with “Quality of Life” (−0.75), indicating that high levels of psychological suffering are intensely associated with a significantly worse perception of overall well-being. “Depression” also showed a notable negative correlation with the “Social Domain” (−0.54), and “Anxiety” with “Quality of Life” (−0.28). It is crucial to clarify that these reported correlations reflect associations within a cross-sectional design and do not imply causality.

The other correlations were considered weak or null, suggesting that, after the diattenuation process, associations between physical and environmental domains and other variables became less evident.

Table S5 in the Supplementary File presents the deattenuated correlation matrix.

## 4. Discussion

This study examined the quality of life (QoL) and mental health of nursing technicians in Brazilian Unified Health System (SUS) emergency settings, a topic of critical relevance, especially given the prolonged and complex impacts of the COVID-19 pandemic on healthcare environments. Our findings highlight the inherent challenges of these demanding work contexts, where resilience and continuous confrontation with stressors are required, aligning with recent research in this area [36].

The profile of the studied sample—predominantly female and with significant professional experience in emergency nursing—reflects the workforce landscape in Brazil and several other countries. This demographic and professional context is crucial for understanding the perceptions and challenges faced by these nursing technicians, whose experiences with the 12 × 36 h regimen and single professional ties typify the reality of emergency services, a reality that was rigorously tested and transformed during and after the global health crisis [37,38].

The QoL analysis, using the WHOQOL-BREF, revealed perception patterns that, although centered on a sample average, exhibited notable heterogeneity in “General Quality of Life.” This variability suggests that, even within a similar professional group, subjective experiences of well-being are diverse, possibly influenced by individual coping factors or micro-work environments. In contrast, the “Environment” domain of QoL proved more homogeneous, indicating a more shared experience regarding environmental aspects that influence the lives of these professionals. Differences in the perception of general and physical QoL between residents of Votuporanga and Fernandópolis indicate that regional contextual factors may play a subtle, but discernible, role in well-being, warranting future investigations into local support infrastructure or organizational culture.

The absence of significant differences in QoL based on variables such as salary range, workload, or multiple professional ties, although contrasting with some literature that points to income and workload as important predictors [39,40], may now underscore the overwhelming influence of the inherent demands of emergency work, further amplified by the prolonged period of pandemic-induced stress. This scenario suggests that the “new normal” in emergency care, characterized by a generalized increase in stress, the potential for loneliness and isolation due to altered protocols, and a sustained state of alert and vigilance, may homogenize the QoL experience across diverse sociodemographic factors. The overarching demand of the role, indelibly redefined by recent global events, appears to have become the predominant stressor. Studies like that of Santos et al. (2025) [36] corroborate this complexity, pointing out that the reliability of certain WHOQOL-BREF domains can affect the detection of associations, suggesting that the measurement of some aspects of QoL can be challenging in high-stress contexts, especially those chronically elevated by the pandemic. The tendency for professionals with a single tie to report better social relationships and for women to show higher scores in this domain may suggest potential implications for balancing multiple work schedules and maintaining support networks in an environment where socialization opportunities are limited and where loneliness may have been intensified.

Regarding mental health, the HADS evaluation showed that, while “Psychological Distress” presented great heterogeneity, “Anxiety” proved to be an experience of reduced variability among professionals. This pattern underscores that anxiety can be a constant and widespread companion, an inherent characteristic, in the daily lives of these technicians, inherent to the unpredictability and high stress of emergency environments [41,42,43]. This finding has relevant clinical implications, as it suggests that a high basal state of anxiety can be the tacit norm in this environment, with the risk of normalizing suffering and contributing to chronic stress and burnout. Corroborating this perspective, a recent study identified a notable prevalence of Minor Mental Disorders (MMD) in nursing professionals, with physical and emotional exhaustion evidenced by the “Reduction of Vital Energy” and “Somatic Symptoms” domains [36]. This persistent anxiety is particularly concerning given the post-pandemic context, where frontline healthcare workers experienced prolonged exposure to severe illness, fatalities, and unprecedented operational demands, contributing to a state of chronic allostatic load conceptually similar to a “Long COVID-19” in terms of their effect on mental health.

The consistency in the absence of statistically significant differences in mental health indicators based on demographic and professional variables reiterates the thesis that the very nature of emergency work, now indelibly shaped by the pandemic’s aftermath, is the primary driver of mental health challenges in this group. Continuous exposure to critical incidents and intense demands, as described by González-García et al. (2025) [42], including altered work dynamics and reduced social interaction opportunities in the post pandemic period, can foster feelings of loneliness and isolation. Although Santos et al. (2025) [36] identified that younger professionals and women might show a higher prevalence of MMD and lower perception of QoL, the female predominance in the sample requires caution in interpreting these gender distinctions.

The study’s correlation analysis revealed significant and clinically pertinent relationships. It was observed that psychological distress is strongly associated with a significantly inferior perception of general QoL, highlighting the intrinsic interdependence between mental health and well-being. This robust relationship is widely supported by research demonstrating the predictive role of QoL on mental health dimensions [36]. Additionally, the negative correlation between depression and the social domain of QoL suggests that depressive suffering can compromise engagement in social relationships, which are essential for support and coping. Anxiety also demonstrated a detrimental impact on QoL, reinforcing the need for interventions that consider mental health as an integral component of well-being. The literature also indicates that the physical demands of nursing contribute to psychological well-being [36], which aligns with the findings of the physical dimension of QoL. These correlations gain even greater significance in a post-pandemic world, where social support networks and informal socialization opportunities were frequently disrupted or restricted, which may have contributed to feelings of increased loneliness and isolation. The intensified need for self-care as a coping mechanism, a direct consequence of pandemic-induced stress, also aligns with these findings.

Although this study did not directly assess resilience, the recent literature, such as the work by Santos et al. (2025) [36], offers a framework for its interpretation. This study indicated that resilience, although not a direct predictor of MMD, maintains a strong positive correlation with QoL. This dynamic suggests that resilience acts as an indirect facilitator of QoL, which, in turn, exerts a more direct influence on the mitigation of MMD [36]. This perspective aligns with the Transactional Model of Stress and Coping [43], which positions resilience as an adaptive process that contributes to a more constructive appraisal of stressors, thereby promoting better QoL and attenuating the negative effects of stress on mental health, essential in a landscape of prolonged stressors and pandemic-transformed environments.

The results of the present study corroborate the literature that highlights burnout as a key factor in nurses’ work QoL, intimately linked to emotional exhaustion and depersonalization [39]. Continuous exposure to high-stress environments, characteristic of emergency nursing, and now exacerbated by the memory and reality of a pandemic, is a known contributor to burnout and reduced QoL [39,43]. This reinforces the importance of strategies that promote supportive work environments, adequate resources, and effective conflict management as essential measures to mitigate stress and improve QoL, aligned with the imperative to address the novel changes brought about by the pandemic in the work environment, as advocated by González-García et al. (2025) [42].

Clinical/Practical Interpretation of Observed Levels: The observed trends, particularly the widespread anxiety and the robust negative correlation between psychological distress and QoL, offer important insights into the long-term psychological state of emergency nursing professionals in the post-COVID-19 era. These findings suggest that nursing technicians may experience a persistent state of chronic allostatic load, which can be interpreted conceptually as a form of “Long COVID-19” affecting their mental health. This condition potentially increases the risk of compassion fatigue, professional disengagement, and care errors, thereby impacting patient safety and quality of care. The minor QoL advantage noted in Votuporanga, though not statistically strong, may point to localized micro-social or institutional factors acting as buffers, suggesting a need for qualitative research to explore such protective practices. The consistency of mental health outcomes across various sociodemographic and professional factors further supports the hypothesis that the intensified demands of emergency work, shaped by pandemic experiences, are the primary stressors. Consequently, effective interventions must go beyond traditional adjustments (e.g., salary, workload) to proactively adapt work environments to new realities, including changes in communication and socialization opportunities, and addressing increased feelings of loneliness and isolation, while also strengthening QoL and individual coping mechanisms in this post-pandemic context. Implementing robust organizational and individual support strategies is crucial for building resilience and mitigating the persistent psychosocial impacts of the pandemic.

### 4.1. Future Perspectives

Considering the limitations of the cross-sectional design and the findings that suggest the complexity of relationships between QoL, mental health, and work factors, future research should explore longitudinal approaches. This would allow for a deeper understanding of the directionality of associations and the temporal dynamics of well-being among nursing professionals, especially under the influence of chronic or acute stressful events. Furthermore, the inclusion of mixed methodologies, combining quantitative data with qualitative data (such as in-depth interviews), could reveal nuances and subjective perspectives that would complement statistical analysis, particularly in the investigation of local contextual factors that may influence QoL and mental health.

Additionally, it would be relevant to further investigate the mediating role of resilience in the relationship between the demands of emergency work and mental health outcomes, as suggested by Santos et al. (2025) [36]. Future studies could also seek to refine QoL measurement instruments, especially those domains that showed low reliability, or develop more specific tools for the emergency nursing context, to capture the complexity of these constructs more accurately. The integration of biological measures of stress, such as biomarkers (e.g., cortisol, heart rate variability), could further offer a more holistic understanding of the psychophysiological mechanisms underlying the mental health challenges faced by these professionals.

### 4.2. Limitations

This study presents some important limitations. Firstly, its cross-sectional design prevents the establishment of causal relationships between variables, making longitudinal studies necessary to understand the temporal dynamics of QoL and mental health among nursing technicians. Secondly, the use of self-report questionnaires, although validated, may be subject to biases such as social desirability, a limitation also pointed out by other research in the area [36]. Additionally, the study was conducted in two specific municipalities in São Paulo state, which may limit the generalization of findings to other geographical and cultural contexts. Finally, the low composite reliability of some WHOQOL-BREF domains (Psychological, Environmental, and Social), observed in other studies [36], suggests that the measurement capacity of these constructs may have been compromised, which could obscure their true predictive or correlational roles in our study’s analysis, impacting more specific conclusions about their independent contributions. Furthermore, the reliance on an online questionnaire distribution and data collection process means there was limited control over participant selection, potentially introducing selection bias and affecting the external validity of the findings.

## 5. Conclusions

In conclusion, this study reaffirms, with robust data, that psychological distress among emergency nursing technicians in the SUS is significantly associated with an unfavorable perception of general QoL and social relationships, with anxiety emerging as a shared and persistent experience. Crucially, demographic and professional characteristics did not prove to be primary differentiators for QoL or mental health, suggesting that the intrinsic and high-stress nature of emergency work, now profoundly influenced by the lasting consequences of the COVID-19 pandemic, acts as the central and predominant stressor.

These findings underscore the critical need to recognize and address the “Long COVID-19” effect (understood as a conceptual framework for prolonged psychosocial impact) on the mental health of this vital workforce, manifesting as increased stress, potential for loneliness, and altered social interactions within the work environment. It is, therefore, imperative that interventions transcend pre-pandemic approaches and specifically seek to strengthen the overall QoL and coping capacities of these essential professionals, proactively adapting work environments to new realities. This includes fostering communication opportunities, combating isolation, and providing robust organizational and individual support strategies to mitigate the amplified psychological impacts inherent in emergency care, thereby promoting both team well-being and the sustained quality and safety of patient care in the post-pandemic era.

### 5.1. What Is Known About the Topic

Nursing professionals, especially those in emergency units, face significant challenges that impact their Quality of Life (QoL) and mental health. These include high operational tension, unpredictable demands, and exposure to traumatic events, which are known to contribute to occupational stress, burnout, and mental health issues like anxiety and depression. Psychological distress, in particular, has been strongly linked to a poorer perception of overall QoL, and resilience is recognized as an important factor in mitigating these negative effects.

### 5.2. What This Paper Adds

This study demonstrates that, for nursing professionals in emergency services within Brazil’s Unified Health System (SUS), the intrinsic nature and demands of emergency work are the central stressors, prevailing over sociodemographic and professional factors such as salary range, workload, and multiple job ties. It reveals significant heterogeneity in general QoL, but low variability in anxiety, suggesting that anxiety is a common and pervasive experience, an inherent characteristic, in this high-complexity environment. The paper reinforces the strong negative correlation between psychological distress and general QoL, and between depression and the social domain. The findings emphasize the need for interventions aimed at strengthening general QoL and coping capacities, as well as adapting the work environment to mitigate the stress inherent in emergency care, going beyond salary adjustments or workload changes to promote both team well-being and the quality of care provided.

## Figures and Tables

**Table 1:** Sociodemographic and Professional Data of the Sample. Values are presented as N (%) for categorical variables and Mean ± Standard Deviation for continuous variables (e.g., Age).

Variables	N = 146
How do you identify in terms of gender?	
Female	125 (86)
Male	21 (14)
Age	36.8 ± 9.3
Where do you reside?	
Fernandópolis	38 (26)
Votuporanga	50 (34)
Is the municipality where you reside the same as where you work?	
No. I need to commute from my municipality of residence.	24 (16)
Yes. I live and work in the same municipality.	122 (84)
Do you have more than one professional tie?	
No. I have only 1 professional tie.	121 (83)
Yes. I have 2 ties.	21 (14)
Yes. I have 3 or more ties.	4 (3)
How long have you been working as a Nursing Technician?	
I have between 6 months and 2 years of experience.	8 (5)
I have between 2 and 4 years of experience.	24 (16)
I have between 4 and 6 years of experience.	16 (11)
I have between 6 and 8 years of experience.	7 (5)
I have between 8 and 10 years of experience.	39 (27)
I have between 10 and 20 years of experience.	52 (36)
What is your work regimen?	
6 h daily	10 (7)
8 h daily.	6 (4)
12 h work per 36 h rest.	130 (89)
What is your salary range?	
I earn between 3 thousand and 4 thousand reais.	98 (67)
I earn more than 5 thousand reais.	48 (33)

Note: N (%); Mean ± standard deviation. Source: Author.

**Table 2:** WHOQOL-BREF Factorial Scores of the Sample. (n = 146; São Paulo—Brazil; 2025).

Variables	Mean	SD	W	*p*-Value	P0	P25	P50	P75	P100
Physical Domain	0.00	0.41	0.97	0.00	−1.25	−0.23	0.07	0.27	1.26
Social Relationships Domain	0.00	0.56	0.96	0.00	−1.76	−0.29	0.06	0.32	1.06
Environment Domain	0.00	0.28	0.99	0.50	−0.96	−0.17	−0.01	0.19	0.68
Quality of Life Domain	0.00	0.83	0.94	0.00	−3.80	−0.40	0.06	0.56	1.52

Note: SD: standard deviation; W: Shapiro–Wilk normality test statistic; *p*-value: from Shapiro–Wilk normality test; P: percentile. Source: Author.

## Data Availability

The original data presented in the study are openly available in Open Science Framework (OSF) at https://osf.io/7q8zw/ (accessed on 9 January 2026), reference number 7Q8ZW.

## Supplementary Materials

The following supporting information can be downloaded at: https://www.mdpi.com/article/10.3390/ijerph23020271/s1, Table S1. HADS Factorial Scores of the Sample. (n = 146; São Paulo—Brazil; 2025); Table S2. Comparative analysis of WHOQOL-BREF scores between nursing technicians residing in Fernandópolis and Votuporanga. (n = 88; São Paulo—Brazil; 2025); Table S3. Comparative analysis of WHOQOL-BREF scores between different salary groups residing in Fernandópolis and Votuporanga. (n = 146; São Paulo—Brazil; 2025); Table S4. Pearson correlation matrix for the correlation between WHOQOL-BREF and HADS. (n = 146; São Paulo—Brazil; 2025); Table S5. Disattenuated Pearson correlation matrix for the correlation between WHOQOL-BREF and HADS. (n = 146; São Paulo—Brazil; 2025); Figure S1. Distribution of the histogram of HAD factor scores for (a) anxiety variable, (b) depression variable, and (c) psychological distress Variable, and Boxplot distribution of HAD factor scores for (d) anxiety variable, (e) depression variable, and (f) psychological distress variable. (n = 146; São Paulo—Brazil; 2025). Source: Author.

## Abbreviations

The following abbreviations are used in this manuscript:

ANS	Anxiety (from HADS)
CEP	Research Ethics Committee
CNES	Cadastro Nacional de Estabelecimentos de Saúde
CNS	National Health Council
DEP	Depression (from HADS)
EM	Environment Domain (from WHOQOL-BREF)
FAMERP	Faculty of Medicine of São José do Rio Preto
FICF	Free and Informed Consent Form
HADS	Hospital Anxiety and Depression Scale
HADS-A	Anxiety Subscale
HADS-D	Depression Subscale
HDI	Human Development Index
IBGE	Instituto Brasileiro de Geografia e Estatística
MMD	Minor Mental Disorders
PD	Psychological Distress (from HADS)
PH	Physical Domain (from WHOQOL-BREF)
QoL	Quality of Life
SO	Social Relationships Domain (from WHOQOL-BREF)
SUS	Unified Health System
WHO	World Health Organization
WHOQOL-BREF	World Health Organization Quality of Life—BREF
