# Supplementary material for: Prolonged Effects on Frontline Caregivers: Occupational Stress and Mental Well-Being in Transformed Healthcare Environments Post-COVID-19
Source: Int J Environ Res Public Health. 2026 Feb 22;23(2):271. doi: 10.3390/ijerph23020271 (PMC12940472; doi:10.3390/ijerph23020271)
Supplement: Supplementary file 1 [file ijerph-23-00271-s001.zip › ijerph-4121788-supplementary.pdf]

Table S1. HADS Factorial Scores of the Sample. (n = 146; São Paulo – Brazil; 2025)

Variables	Mean	SD	W	p-value	P0	P25	P50	P75	P100
**Anxiety**	0.00	0.23	0.98	0.04	-0.53	-0.17	-0.02	0.12	0.75
**Depression**	0.00	0.46	0.93	0.00	-1.15	-0.25	-0.06	0.15	1.77
**Psychological Distress**	0.00	0.67	0.98	0.09	-1.26	-0.51	-0.07	0.49	1.71

Note: SD: Standard deviation; W: Shapiro-Wilk normality test statistic; p-value: from Shapiro-Wilk normality test; P: Percentile. Source: Author.

Table S2. Comparative analysis of WHOQOL-BREF scores between nursing technicians residing in Fernandópolis and Votuporanga. (n = 88; São Paulo – Brazil; 2025)

Variables	Fernandópolis N = 33[1]	Votuporanga N = 50[1]	p-value[2]	ESD [95% CI]
**Physical**	-0.06 [0.58]	0.16 [0.49]	0.055	0.211 [0.006; 0.41]
**Social Relationships**	0.10 [0.69]	0.07 [0.65]	0.996	0.001 [-0.21; 0.04]
**Environment**	-0.01 [0.40]	0.08 [0.40]	0.885	0.016 [-0.2; 0.07]
**Quality of Life**	-0.19 [0.62]	0.23 [0.97]	0.045	0.221 [0.02; 0.42]

[1]*Median [IQR];* [2]*Mann-Whitney U test; ESD: R-biserial effect size. Source: Author.*

Table S3. Comparative analysis of WHOQOL-BREF scores between different salary groups residing in Fernandópolis and Votuporanga. (n = 146; São Paulo – Brazil; 2025)

Variables	I earn between 3 thousand and 4 thousand reais. N = 98[1]	I earn more than 5 thousand reais. N = 48[1]	p-value[2]	ESD [95% CI]
**Physical Domain**	0.05 [0.52]	0.12 [0.34]	0.437	0.064 [-0.08; 0.17]
**Social Relationships Domain**	0.11 [0.60]	-0.04 [0.73]	0.124	0.128 [-0.03; 0.27]
**Environment Domain**	0.03 [0.35]	-0.02 [0.36]	0.234	0.099 [-0.05; 0.22]
**Quality of Life Domain**	0.03 [0.90]	0.23 [1.07]	0.304	0.085 [-0.07; 0.2]

[1]Median [IQR]; [2]Mann-Whitney U test; ESD: R-biserial effect size. Source: Author.

Table S4. Pearson correlation matrix for the correlation between WHOQOL-BREF and HADS. (n = 146; São Paulo – Brazil; 2025)

Variables	1.	2.	3.	4.	5.	6.	7.
**1. PH**	1.00	-	-	-	-	-	-
**2. SO**	0.00	1.00	-	-	-	-	-
**3. EM**	0.00	0.00	1.00	-	-	-	-
**4. QoL**	0.00	0.00	0.00	1.00	-	-	-
**5. ANS**	-0.03	-0.05	0.05	-0.18 *	1.00	-	-
**6. DEP**	0.00	-0.23 **	0.00	-0.14	0.00	1.00	-
**7. PD**	0.00	-0.10	-0.07	-0.64 ***	0.00	0.00	1.00

Note: Values in brackets indicate 95% confidence interval for each correlation. The confidence interval is a plausible range of population correlations that could have caused the sample correlation [35]. *PH: Physical Domain; SO: Social Domain; EM: Environmental Domain; QoL: Quality of Life; ANS: Anxiety; DEP: Depression; PD: Psychological Distress. * indicates p < .05. ** indicates p < .01. *** indicates p < .001. Source: Author.*

Table S5. Disattenuated Pearson correlation matrix for the correlation between WHOQOL-BREF and HADS. (n = 146; São Paulo – Brazil; 2025)

Variables	PH	SO	EM	QoL	ANS	DEP	PD
**PH**	1.00	0.00	0.00	0.00	-0.06	-0.01	0.00
**SO**	0.00	1.00	0.00	0.00	-0.13	-0.54	-0.19
**EM**	0.00	0.00	1.00	0.00	0.09	0.00	-0.11
**QoL**	0.00	0.00	0.00	1.00	-0.28	-0.20	-0.75
**ANS**	-0.06	-0.13	0.09	-0.28	1.00	0.00	0.00
**DEP**	-0.01	-0.54	0.00	-0.20	0.00	1.00	0.00
**PD**	0.00	-0.19	-0.11	-0.75	0.00	0.00	1.00

Note: PH: Physical Domain; SO: Social Domain; EM: Environmental Domain; QoL: Quality of Life; ANS: Anxiety; DEP: Depression; PD: Psychological Distress. Source: Author.

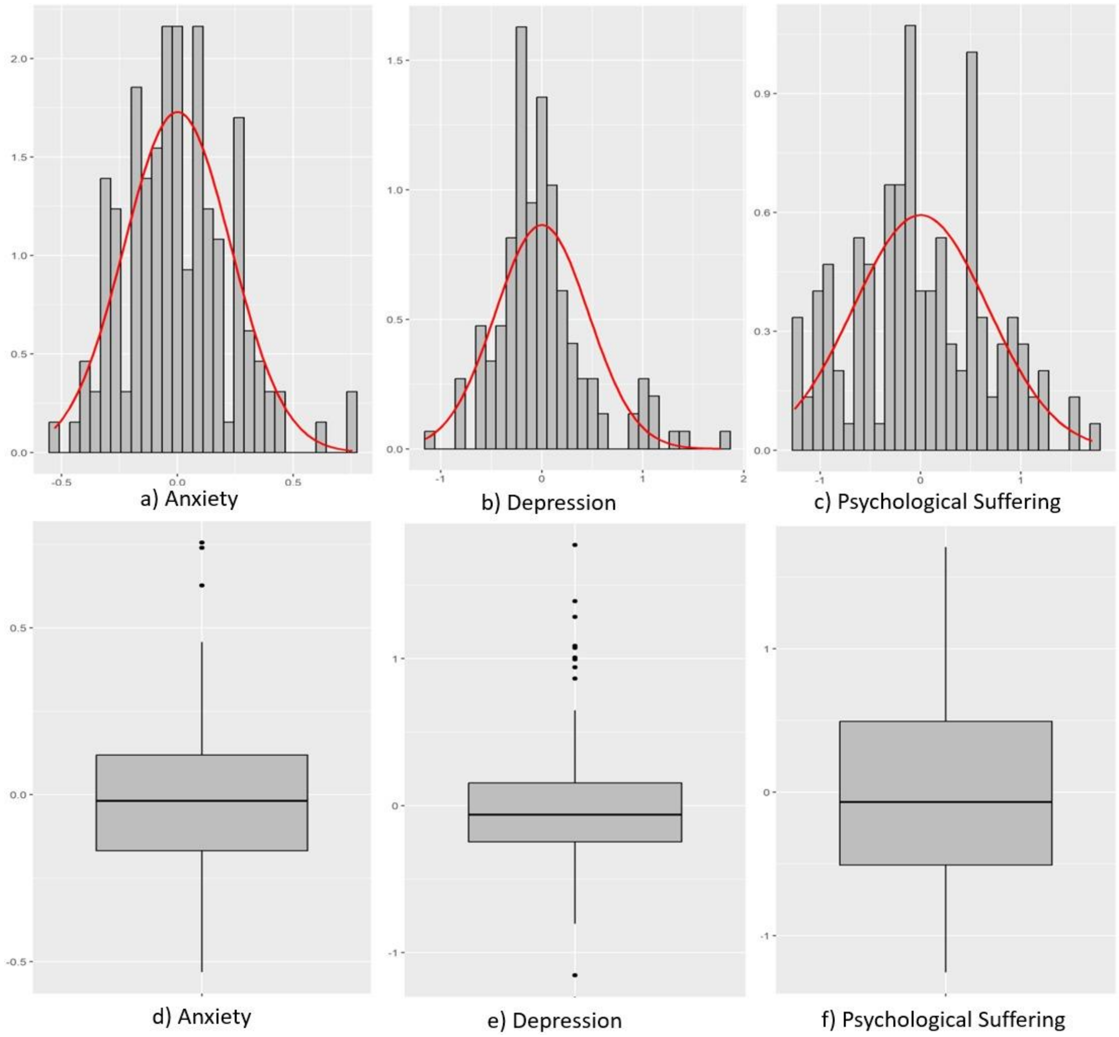

Figure S1. Distribution of the histogram of HAD factor scores for: a) Anxiety Variable; b) Depression Variable; c) Psychological Distress Variable; and Boxplot distribution of HAD factor scores for: d) Anxiety variable; e) Depression variable; f) Psychological Distress variable. (n = 146; São Paulo – Brazil; 2025). Source: Author.
